# Scale of Death Anxiety (SDA): Development and Validation

**DOI:** 10.3389/fpsyg.2017.00858

**Published:** 2017-05-31

**Authors:** Wei Cai, Yung-lung Tang, Song Wu, Hong Li

**Affiliations:** ^1^Research Centre of Brain Function and Psychological Science, Shenzhen UniversityShenzhen, China; ^2^Key Laboratory of Behavioral Science, Institute of Psychology, Chinese Academy of SciencesBeijing, China; ^3^Faculty of Psychology, Southwest UniversityChongqing, China; ^4^Shenzhen Key Laboratory of Affective and Social Cognitive Science, Shenzhen UniversityShenzhen, China; ^5^Center for Language and Brain, Shenzhen Institute of NeuroscienceShenzhen, China

**Keywords:** death anxiety, SDA, confirmatory factor analysis, scale development, Dysphoria, Death Intrusion, Fear of Death, Avoidance of Death

## Abstract

This study developed and validated a new measure to assess the death anxiety (i.e., Scale of Death Anxiety, SDA) on an individual’s somatic, cognitive, emotional, and behavioral reactions from a symptomatic perspective in Chinese youth samples. Following a systematic process, a four-factor structure of the SDA was identified through principle components analysis and confirmatory factor analysis that revealed four aspects of death anxiety: Dysphoria, Death Intrusion, Fear of Death, and Avoidance of Death. The results of this study indicate that the SDA has a clear factor structure and good psychometric properties. The SDA supports death anxiety as a multidimensional construct, and the foundational role of fear of death in the generation of death anxiety. This scale is valuable and beneficial to research on death anxiety. This study makes a significant contribution to the literature because the SDA is the first assessment of death anxiety to include the constructs of dysphoria and somatic symptoms. And the potential clinical practice of the SDA was discussed.

## Introduction

All but Death, can be Adjusted. – Emily Dickinson

As Emily Dickinson wrote, death is the one thing that nobody can avoid; it is also a powerful motivator of human behavior (Freud, 1914). The inevitability and unpredictability of death cause people to feel horror, and this fear of death is a fundamental source of anxiety (Yalom, 1980). Death anxiety derives from death awareness and is decreased through distal terror management defenses (Pyszczynski et al., 1999). Meanwhile, the mismanagement of death anxiety aggravates symptoms of mental disorders (Arndt et al., 2005; Strachan et al., 2007). Due to the theoretical and clinical importance of death anxiety, many questionnaires (see **Table 1**) have been developed to assess this construct since 1970.

**Table 1:** Summary of currently available death anxiety measures.

Measure title	Items	Rating scale	Factors	Sample (*N*)
1 Death Anxiety Scale (DAS) Templer, 1970	15	True–false	Unidimensional at first, but diverse factor structures were found across various samples	141 College students
2 Revised Death Anxiety Scale (RDAS) Thorson and Powell, 1994	25	True–false/5-point	Diverse factor structures across various samples	Various
3 Death Anxiety Scale-Extended (DASE) Templer et al., 2006	51	True–false	1. Externally caused deaths, 2. The thought of death, 3. Excruciating pain, 4. Fear of surgery, 5. The image of death, 6. Death proximity, 7. Presence of death, 8. Death anxiety denial, 9. Dreams of death, and 10. Death thoughts	940 Age range from 11 to 96
4 Multidimensional Death Anxiety Scale (MDAS) Nelson and Nelson, 1975	20	Likert scale	1. Death avoidance, 2. Death fear, 3. Death denial, and 4. Reluctance to interact with the dying	135 Students 1279 Residents
5 Death Anxiety Questionnaire (DAQ) Conte et al., 1982	15	3-point, not at all (0) – somewhat (1) – very much (2)	1. Fear of the unknown, 2. Fear of suffering, 3. Fear of loneliness, and 4. Fear of personal extinction	230 Graduate students
6 Chinese Death Anxiety Inventory (CDAI) Wu et al., 2003	23	5-point, degree of agreement	1. Death and dying anxiety, and 2. After–death anxiety	282 Chinese college students
7 Arabic Scale of Death Anxiety (ASDA) Abdel-Khalek, 2004	20	5-point, no (1) – very much (5)	1. Fear of dead people and tombs, 2. Fear of postmortem events, 3. Fear of lethal disease, and 4. Death preoccupation.	1636 Arabic undergraduates
8 Death Anxiety Inventory (DAI) Tomás-Sábado and Gómez-Benito, 2005	20	True–false/6-point agreement	1. Externally generated death anxiety, 2. Meaning and acceptance of death, 3. Thoughts about death, 4. Life after death, and 5. Brevity of life.	2039 Spanish

As shown in **Table 1**, the development of measures of death anxiety reflects an evolution of the definition of death anxiety, which has evolved over time from a unidimensional construct to a multidimensional one. Templer (1970) approached death anxiety as a subjective painful experience common to everyone and originally developed the 15-item self-report Death Anxiety Scale (DAS), which has been widely used. However, the DAS has subsequently been shown to have a diverse factor structure by Templer et al. (2006) and other researchers (Lonetto et al., 1979; Martin, 1982; Schell and Zinger, 1984; Lonetto and Templer, 1986). Factor structures have also varied with the source and size of the sample (Lonetto et al., 1979). Considering evidence that not all individuals feel anxiety about death (Neimeyer and Van Brunt, 1995), increasingly researchers have conceptualized death anxiety as a multidimensional construct.

A review of currently available scales of death anxiety (in English or Chinese), for instance, the Death Anxiety Scale (Templer, 1970), Revised Death Anxiety Scale (Thorson and Powell, 1994), Death Anxiety Scale-Extended (Templer et al., 2006), Multidimensional Death Anxiety Scale (Nelson and Nelson, 1975), Death Anxiety Questionnaire (Conte et al., 1982), Chinese Death Anxiety Inventory (Wu et al., 2003), Arabic Scale of Death Anxiety (Abdel-Khalek, 2004), and Death Anxiety Inventory (Tomás-Sábado and Gómez-Benito, 2005), revealed some issues to be resolved. First, current scales about death anxiety tend to measure apprehension about specific death-related events rather than physical reactions. Specifically, they assessed an individual’s cognition and emotion about death or specific things related to death, such as surgery, loneliness, personal extinction, and so on (e.g., Templer, 1970; Nelson and Nelson, 1975; Conte et al., 1982; Abdel-Khalek, 2004; Templer et al., 2006). It is known that physical reaction is a core feature of the nature of anxiety, that is “*the apprehensive anticipation of future danger or misfortune accompanied by a feeling of dysphoria or somatic symptoms of tension, when there is no true threat*.” (Vermetten et al., 2002); nevertheless, few current measurements of death anxiety include assessments about individuals’ physical reactions. In addition, the internal consistency reliability and factor structures of the scales have been shown to be unstable across different samples (Lonetto and Templer, 1986; Thorson and Powell, 1990, 1994), because a single scale cannot capture all of the potential death-related events without being overly long.

Second, current scales give greater weight to fear of death and do not clearly distinguish it from death anxiety. Fear of death and death anxiety are two distinct theoretical constructs (Lehto and Stein, 2009). On one hand, fear and anxiety share similar emotional and behavioral consequences, such as avoidance of places, events, or things that cause fear or anxiety. On the other hand, fear emphasizes negative emotional reactions to visible, specific events or true threats, whereas anxiety emphasizes negative reactions to non-specific, potential, and distal threats or stimulations with vigilance and arousal (Vermetten et al., 2002). In addition, these two states may result in two different clinical consequences (Blanchard et al., 2011). Yalom (1980) thought that fear of death was the foundation of death anxiety, such that everyone would feel fear of death, but only some would experience death anxiety. Therefore, considering the nature of anxiety, the assessment of death anxiety should not only include the shared features of fear and anxiety, which have been accepted by most researchers (Nelson and Nelson, 1975; Nelson, 1978), but also reflect unique features of anxiety, such as avoiding death-related things or events as well as dysphoria and somatic reactions related to anxiety.

Third, apart from the fear of death, some existing death anxiety measures include antecedent components that influence death anxiety, such as “externally generated death anxiety” and general healthy statements (e.g., Tomás-Sábado and Gómez-Benito, 2005; Templer et al., 2006). Furthermore, some death anxiety scales include factors reflecting an individual’s attitude toward death (e.g., Nelson and Nelson, 1975; Tomás-Sábado and Gómez-Benito, 2005). These factors are important in terms of researchers understanding death anxiety, but are less related to the nature and features of death anxiety itself.

Therefore, a new scale that measures all these components of death anxiety was proposed and developed in the current study to resolve the above existed issues. The current study aimed to, firstly, develop a new inventory for death anxiety, the Scale of Death Anxiety (SDA), as a new measure of the death anxiety on individuals’ cognitions, emotions, behavioral and somatic reactions from a symptomatic perspective; and secondly, validate the SDA in a Chinese youth sample.

Consistent with previous literature, the current study proposed death anxiety to be a multidimensional construct. Based on the nature of anxiety and death, the SDA not only measures psychological components, but also somatic symptoms and dysphoria. The SDA does not assess an individual’s responses to concrete death-related things and situations (e.g., tombs, surgery, and so on), but rather focuses on general reactions to and impacts of death itself. Specifically, we proposed a four-dimension model of the death anxiety in the current study: *Dysphoria, Death Intrusion, Fear of Death*, and *Avoidance of Death*. Dysphoria emphasizes the somatic component of the death anxiety (DA), referring to feelings of being tired, upset, and emotionally isolated when thinking of death. Death intrusion emphasizes the cognitive component of the DA, referring to intrusive nightmares, imagery, and thoughts related to one’s own death. Fear of death emphasizes the emotional component of the DA, referring to feelings of being scared of death with emotional and somatic symptoms. Finally, avoidance of death emphasizes the behavioral component of the DA, referring to the avoidance of thoughts, situations, events, and experiences related to death. Overall, two studies were conducted to explore and identify the latent structure of the scale (Study 1) and confirm the structure (Study 2). Study 2 also explored the criterion validity of the SDA by using the Beck Depression Inventory, Trait Anxiety Subscale, Impact of Event Scale-Revised, and Subjective Happiness Scale. Both studies were conducted in Chinese, to enrich the death anxiety literature and aid in understanding the Chinese experience of death anxiety.

## Study 1: Scale Construction and Development

### Materials and Methods

#### Participants

Three hundred and fifty-three participants from high schools and universities in the north of China (i.e., Shanxi) and southwest of China (i.e., Chongqing) were invited to take part in this study using a convenience sampling method, and 325 participants (150 males, 175 females) completed the whole questionnaire. The proportion of valid questionnaires was 92.1%. The age range was from 12 to 23, *Median* = 17.00, *Mean* (*M*) = 17.01, *SD* = 3.06. Eighty-nine percent of participants (*n* = 288) reported no religion, 6% (*n* = 21) were Buddhist, 2% (*n* = 6) were Christian, 2% (*n* = 5) were other, 1% (*n* = 4) were Muslim, and 1 did not report. The education level of the sample was as follows: 117 college, 102 senior high school, and 106 junior high school. No selection criteria were used.

#### Item Generation of the Pilot Death Anxiety Questionnaire

There were four steps to generate the items of the pilot death anxiety questionnaire, which are described as follows.

##### Concept definition

Previous theoretical and empirical literature discussing death anxiety, anxiety, and related constructs (e.g., fear of death, death attitudes, and anxiety disorders) and currently available measures of death anxiety were reviewed to generate items. Considering the behavioral consequences and nature of anxiety, *Death Anxiety* was defined as the state in which an individual experiences physical symptoms of being upset and nervous, and dreaded feelings of worry and fear related to one’s own death and dying generated by an imagined threat to one’s existence. Specifically, it included four dimensions as follows: (1) physiological nervous reactivity; (2) recurrent thoughts about death and dying or death-related events; (3) feeling worry and fear when thinking about one’s own death or dying; and (4) avoidance of thoughts and events associated with death and dying.

##### Item generation

To generate questionnaire items that reflected the content of death anxiety, the following instruments were reviewed: Death Anxiety Scale (Templer, 1970), Death Anxiety Scale-Extended (Templer et al., 2006), Revised Death Anxiety Scale (Thorson and Powell, 1994), Multidimensionality of Death Anxiety (Nelson and Nelson, 1975; Nelson, 1978), Death Anxiety Questionnaire (Conte et al., 1982), Chinese Death Anxiety Inventory (Wu et al., 2003), Death Anxiety Inventory (Tomás-Sábado and Gómez-Benito, 2005), Collett–Lester Fear of Death Scale (Collett and Lester, 1969; Lester, 1990), Arabic Scale of Death Anxiety (Abdel-Khalek, 2004), and the physiological items of anxiety from the Self-Rating Anxiety Scale (Zung, 1971). If an item was reflected one of the four dimensions that we described above, it was included in the potential items pool of this study. Following this process, 44 potential items were created to reflect the construct of death anxiety. And all of these potential items or original Chinese form are reported in detail in the Supplementary Data Sheet 2 in the Supplementary Material.

##### Experts’ review of items

A panel of five experts in death research and/or psychological counseling (two associate psychology professors and three psychology graduate students, including the second author of the current study) discussed, evaluated, and modified the potential items based on three principles: (1) whether the definition and theoretical dimensions of death anxiety were reasonable; (2) whether the items were consistent with the content of death anxiety; and (3) whether the wording of the items was accurate. Each professional evaluated the items independently. Two items were removed because they were suggested to delete by one or more professional (i.e., one vote veto principle). More than two experts thought three items may not suitable for reflecting death anxiety (i.e., experts’ agreement rate was less than 3/5), they were then removed. And the words of six items were discussed and modified until all five professionals agreed it clear. To ensure the items reflected an individual’s recent state regarding death and dying, all began with the phrase “In the past month, I have often…” For items that did not include the wording “death” or “dying,” an extra phrase “whenever thinking of death” was added at the beginning. For example, recurrent thoughts about death was assessed with the statement “In the past month, I have often thought of my own death,” and physiological tension was assessed with the statement “In the past month, whenever thinking of death, I have often felt tense.” Finally, 39 items in total were retained in this phase, and randomly ordered to create a pilot questionnaire.

##### Creation of the pilot questionnaire

A 5-point Likert response scale (1 = Strongly disagree, 2 = Disagree, 3 = Sometimes disagree, sometimes agree, 4 = Agree, 5 = Strongly agree) was used not only to sensitively distinguish individuals’ responses regarding their feelings and perceptions, but also to reduce their cognitive load and make it easier to respond. The following instruction was used: “*Here is a list of statements about how an individual may feel and the perceptions an individual may have when thinking of death and dying. Please think about your feelings and physiological reactions to death and dying in the past month, consider how well each statement relates to you, and indicate your answer from 1 (Strongly disagree) to 5 (Strongly agree).”* To ensure both the wording and content were correct and non-ambiguous, 30 participants different from the main sample were recruited and volunteered to finish the 39-item pilot questionnaire. No misunderstanding of the statements was indicated.

#### Procedure

First, the study procedure was explained to participants to make sure all of them understood. Then, written and oral informed consent was obtained from all participants. For participants under 18 years old, we obtained written informed consent from their parents or guardians first. Second, participants completed a 39-item pilot questionnaire about death anxiety and some demographic questions. Both Study 1 and Study 2 were approved by the Research Ethics Committee of the Faculty of Psychology, Southwest University, Chongqing. We also complied with the ethical standards in the treatment of human subjects.

#### Data Analysis

First, item-total statistics were used to test whether all items were consistent with the scale. Inconsistent items were removed based on the results. Second, Kaiser–Meyer–Olkin (KMO) and Bartlett’s test of sphericity were used to test whether the data were appropriate for factor analysis. Third, a series of principle components analysis (PCA) was used to explore the latent structure of the death anxiety item set and item reduction using SPSS 21.0. A promax rotation (Kappa value of 4) method was chose due to the nature of the items (Fabrigar et al., 1999). The criteria for dimensions and item reduction (Worthington and Whittaker, 2006) were as follows: (1) eigenvalues greater than 1; (2) factors contain three or more items; (3) items load strongly (>0.40) onto factors; (4) items do not cross-load onto two or more factors.

### Results and Discussion

There were no missing data. Based on the item analysis, one item (item 35: “feeling hand is dry and warm”) was removed because of its negative association with the total score, *r* = -0.35. The remaining 38 items (*r*s > 0.20) were moved to the next analysis. The KMO measure (=0.92) and the Bartlett’s test (χ^2^ = 5473.40, *p* < 0.001) showed that the sample was adequate for factor analysis.

Using an eigenvalue greater than 1, PCA identified nine factors with a cumulative extraction sums of squared loadings of 61.6%. However, based on the results of the promax rotation, three factors were weak: one factor only contained one item which cross-loaded (>0.31) onto two other factors; another two factors contained three or four items, some of which cross-loaded (>0.32) onto other factors that had less than two items per factor. Those three factors were dropped from the solution. According to the criteria for items reduction, two items were removed because their coefficients were below 0.4, and three items were removed because they cross-loaded onto other factors (>0.32). Following this process, 25 items were retained.

**FIGURE 1:**
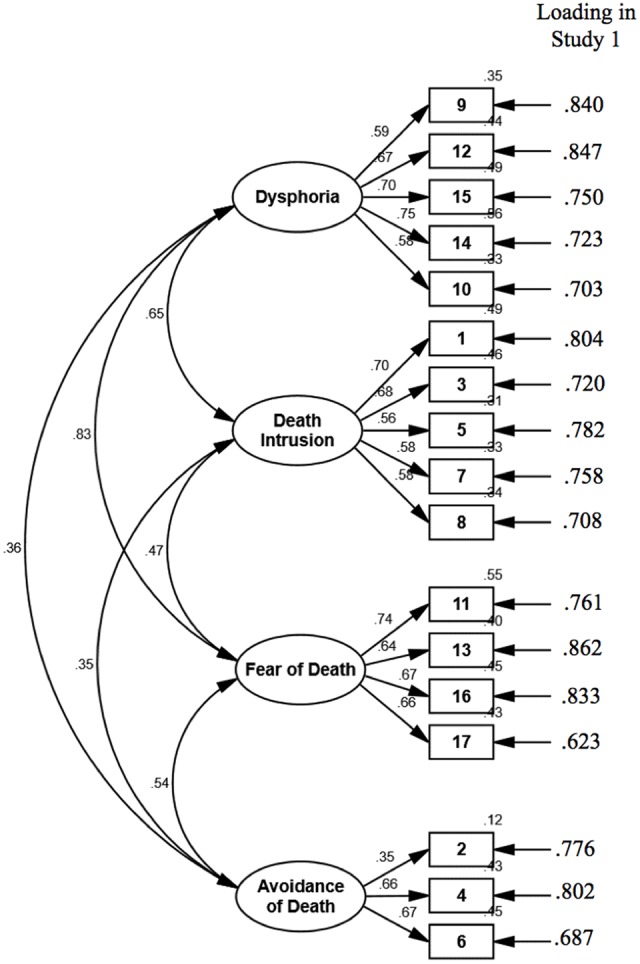
**Confirmatory factor analysis in Study 2, and rotated (promax) factor loadings in Study 1 with principal component analysis**.

Using the same process, we repeated the factor analysis three more times to explore the best fitting latent structure of death anxiety and to reduce the questionnaire length. In the first round, two items were removed because of one coefficient below 0.4, and one cross-loading onto another factor (>0.31). In the second round, three items were removed because of their strong cross-loadings (>0.34) onto other factors. In the third round, one factor was removed because it only contained two items, and one item was removed because of its cross-loading (>0.34) onto another factor. Ultimately, four factors remained with 17 items, and the cumulative extraction sums of squared loadings was 62.1%. The remaining four factors all contained at least three items, and the loading of each item was more than 0.62 (see **Figure 1**). The items of the original Chinese form are in **Appendix**.

As **Table 2** shows, the four factors were defined as follows: Dysphoria (five items, *M* = 2.02, *SD* = 1.02, skewness = 0.86, kurtosis = -0.06), Death Intrusion (five items, *M* = 1.65, *SD* = 0.82, skewness = 1.63, kurtosis = 2.61), Fear of Death (four items, *M* = 1.83, *SD* = 0.92, skewness = 1.30, kurtosis = 1.20), and Avoidance of Death (three items, *M* = 2.13, *SD* = 0.99, skewness = 0.69, kurtosis = -0.18). These four factors were consistent with the original definition of death anxiety. Before data collection with a new sample, a confirmatory factor analysis (CFA) with maximum likelihood method was run with the current sample to further explore the strength of the proposed four-factor structure model with 17 items. The model fit well (CFI = 0.93, RMSEA = 0.067, 90% CI = 0.057–0.077, SRMR = 0.062), which supported the proposed model.

**Table 2:** Description of factors identified in Study 1.

Name of factor	Description	Example item
Dysphoria	Feel tired, upset, and emotionally isolated when thinking of death	“*In the past month, whenever thinking of death, I have often gotten upset.”*
Death Intrusion	Intrusive nightmares, imagery, and thoughts related to one’s own death	“*In the past month, I have often thought of my own death.”*
Fear of Death	Feel scared of death with emotional and somatic symptoms.	“*In the past month, whenever thinking of death, I have often felt scared.”*
Avoidance of Death	Avoidance of thoughts, situations, events, and experiences related to death	“*In the past month, I have often avoided thoughts or topics related to death.”*

Following an appropriate process, Study 1 resulted in a 17-item scale with four factors. The proposed model measured somatic dysphoria, which was not present in previous death anxiety instruments, as well as emotional (i.e., Fear of Death), cognitive (i.e., Death Intrusion), and behavioral (i.e., Avoidance of Death) components of death anxiety. The skewness and kurtosis for all factors were within a tolerable range for assuming a normal distribution (±3; D’Agostino et al., 1990). However, this four-factor structure of death anxiety was based on one sample. Therefore, Study 2 was conducted to re-test and confirm the factor structure in a replication sample.

## Study 2: Validation

To confirm the four-factor structure and test the validity and reliability of the 17-item SDA, Study 2 collected new data from a replication sample. A CFA was conducted to test the four-factor model of death anxiety. Furthermore, sample studies indicate a positive relationship between death anxiety and general anxiety or depression (Abdel-Khalek, 2001), and greater impacts of stressful events (such as illness, experiences with death and dying, unpredictable environments, and so on) (see Lehto and Stein, 2009 for a review), but a negative relationship between death anxiety and subjective well-being (e.g., Chaiwutikornwanich, 2015). Thus, general anxiety (assessed by the Trait Anxiety Subscale of the State-Trait Anxiety Inventory), general depression (assessed by the Beck Depression Inventory-II), impacts of stressful events (assessed by the Impact of Event Scale-Revised), and subjective well-being (assessed by the Subjective Happiness Scale) were used to evaluate the criterion validation of the SDA.

### Materials and Methods

#### Participants

A sample of 375 participants from high schools and universities in the north of China (i.e., Shanxi) and southwest of China (i.e., Chongqing) took part in this study, and 342 participants (116 males, 226 females) completed all questionnaires. The proportion of valid questionnaires was 91.2%. The age range was from 13 to 23, *Median* = 19.00, *M* = 18.21, *SD* = 2.24. Ninety percent of participants (*n* = 305) reported no religion, 5.6% (*n* = 19) were Buddhist, 2% (*n* = 7) were Christian, 1.5% (*n* = 5) were Catholic, 0.6% (*n* = 2) were Muslim, and 1.3% (*n* = 4) did not report. The education level of the sample was as follows: 188 college, 87 senior high school, and 67 junior high school. No selection criteria were used.

Although the replication sample was drawn from the same locations as the sample in Study 1, they had no overlap. To test a model, the subjects-to-parameters ratio could not be lower than 5:1 (Bentler and Chou, 1987), and total number of subjects needed to be over 200 (Boomsma, 1985). The replication sample (*n* = 342) in Study 2 reached an 8.5:1 subjects-to-parameters ratio, which was appropriate for testing a model with 40 parameters, that is 17 factor loadings, 17 error variances, and 6 factor correlations.

#### Procedure and Measures

Researcher introduced and explained the procedure of the study to make sure all participants understood. Written and oral informed consent were then obtained from all participants; for participants under 18 years old, we obtained written informed consent from their parents or guardians. In addition to the SDA, to evaluate criterion validity, participants were required to complete scales that assessed trait anxiety, depression, impact of events, and subjective happiness. Finally, to measure the test–retest reliability, a subset of participants (*n* = 74) completed the SDA a second time 7 days later and were given a gift valued at about 5 *RMB*.

##### Beck Depression Inventory (BDI-II, Beck et al., 1996)

Beck Depression Inventory was chose to assess individual’s depressive statement in the past 2 weeks. This is a 21-item scale with a 4-point response scale from 0 to 3 (e.g., “Sadness, 0 = *I do not feel sad*. 1 = *I feel sad much of the time.* 2 = *I am sad all the time*. 3 = *I am so sad or unhappy that I can’t stand it*.”). A higher score indicates greater depression. The Cronbach’s alpha of this scale in the current study is 0.88. One participant failed to finish the BDI-II, but completed the SDA and other measures. Therefore, the total number of participants for analysis with the BDI-II was 341. We hypothesized that the overall SDA and four dimensions of SDA would be positively associated with the BDI-II.

##### Impact of Event Scale – Revised (IES-R, Weiss, 2007)

This is a 22-item self-evaluation scale that measures individual’s experience and responses to a specific stressful event in life. It is composed of three subscales that reflect three typical response sets of intrusion (e.g., “I had trouble staying asleep”), avoidance (e.g., “I felt as if it hadn’t happened or wasn’t real”), and hyperarousal (e.g., “I was jumpy and easily startled”). Individuals are required to evaluate the degree of distress each statement for themselves during the past 7 days. They need to respond on a 5-point scale from 0 (*not at all*) to 4 (*extremely*). The Cronbach’s alpha of intrusion, avoidance, hyperarousal and the whole IES-R in the current sample is 0.83, 0.81, 0.76, and 0.91, respectively. We hypothesized that SDA and its four dimensions would be positively associated with all three subscales and the total subjective stress score of IES-R.

##### Trait Anxiety (TA, Spielberger et al., 1983)

A 20-item subscale assessed individual’s trait anxiety of the State-Trait Anxiety Inventory – Form Y was used. Items are rated from 1 (*Almost never*) to 4 (*Almost always*), and include: “I am content; I am a steady person,” and “I worry too much over something that really doesn’t matter” for example. A higher score indicates greater trait anxiety. The Cronbach’s alpha in the current study is 0.80. We hypothesized that SDA and its four dimensions would be positively associated with the trait anxiety.

##### Subjective Happiness Scale (SHS, Lyubomirsky and Lepper, 1999)

This is a 4-item self-evaluation scale that measures individual’s subjective happiness based on a subjectivist approach. Individuals need to rate item from 1 to 7, such as “In general, I consider myself: 1 = n*ot a very happy person* to 7 = *a very happy person*,” and “Some people are generally not very happy. Although they are not depressed, they never seem as happy as they might be. To what extend does this characterization describe you? 1 = *not at all* to 7 = *a great deal*” (reversed). Higher scores indicate greater happiness. The Cronbach’s alpha in the current study is 0.61. We hypothesized that the overall SDA and its four dimensions would be negatively associated with the subjective happiness.

#### Data Analysis

Firstly, CFA was run using AMOS 20.0 on the 17-item SDA. Specifically, a maximum likelihood model was used to test the structural model. The criteria for indexes that used to evaluate the goodness of fit of the model were as follows (see Hu and Bentler, 1999): chi-square statistics was not significant (*p* > 0.05). Or, if the chi-square statistics was significant (*p* < 0.05), then see the following three indices: comparative fit index (CFI) of 0.90 or more, root-mean-square error of approximation (RMSEA) of 0.08 or less, and standardized root-mean-square residual (SRMR) of 0.08 or less. Then, descriptive statistical analysis was used to examine the mean, standard variation, skewness, and kurtosis of the four factors. Next, correlational analysis was conducted on using SPSS 21.0 to assess criterion validity and the test–retest reliability. Finally, independent *t*-test was used to test the effects of gender (male vs. female) and religion (religious vs. non-religious) on SDA, and the relationship between age and SDA was tested through correlational analysis.

### Results and Discussion

#### Confirmatory Factor Analysis (CFA)

There were no missing data. To confirm the four-factor structure model developed from Study 1, CFA with maximum likelihood method was conducted. The results revealed a good fit to the data of Study 2, χ^2^ = 302.72, χ^2^/*df* = 2.73, *p* < 0.001, CFI = 0.90, RMSEA = 0.07, 90% CI = 0.065–0.084, SRMR = 0.059. The standardized coefficients of each path are shown in **Figure 1**. Descriptive analysis showed that the distributions were approximately normal (D’Agostino et al., 1990) for the overall SDA (*M* = 2.12, *SD* = 0.65, skewness = 0.46, kurtosis = -0.29) and its four components, that is, Dysphoria (*M* = 2.22, *SD* = 0.91, skewness = 0.49, kurtosis = -0.31), Death Intrusion (*M* = 1.75, *SD* = 0.72, skewness = 1.22, kurtosis = 1.69), Fear of Death (*M* = 2.22, *SD* = 0.96, skewness = 0.45, kurtosis = -0.59), and Avoidance of Death (*M* = 2.48, *SD* = 0.87, skewness = 0.15, kurtosis = -0.42).

#### Reliability

In terms of internal consistency reliability, Cronbach’s α for the whole SDA was good, at α = 0.86. In terms of the four factors, Cronbach’s α was 0.80 for Dysphoria, 0.78 for Death Intrusion, 0.77 for Fear of Death, and 0.57 for Avoidance of Death. These results demonstrated that the items were internally consistent.

Regarding test–retest reliability, 74 participants from the current sample completed the SDA twice, with a time interval of 7 days. Cronbach’s α of the SDA for these participants was 0.88 the first time, and 0.86 the second time. Pearson’s correlation coefficient was significant, *p* < 0.001, and *r* = 0.69, 95% CI = [0.54, 0.81] for the overall SDA, *r* = 0.60, 95% CI = [0.45, 0.72] for Dysphoria, *r* = 0.59, 95% CI = [0.45, 0.72] for Death Intrusion, *r* = 0.57, 95% CI = [0.38, 0.72] for Fear of Death, and *r* = 0.55, 95% CI = [0.33, 0.71] for Avoidance of Death. This result indicated good stability of responses to the SDA across time.

#### Criterion Validation

Pearson correlational analysis was conducted to explore the association between the SDA and other criterion measures. Results showed expected associations with a significant moderate and low coefficient of correlation (*r*s ranged from -0.24 to 0.48, *p* < 0.001), providing evidence that the overall SDA was measuring relatively distinct death anxiety and could predict other important behavioral and psychological symptoms. As shown in **Table 3**, the overall SDA was significantly positively associated with an individual’s depression (*r* = 0.40), trait anxiety (*r* = 0.39), and impact of traumatic or stressful events (*r*s = 0.41–0.48), and negatively associated with an individual’s subject happiness (*r* = -0.24), *p* < 0.001. Regarding the four dimensions, all of them presented expected associations with a significant coefficient of correlation as did the overall SDA (*r*s ranged from -0.12 to 0.48, *p* < 0.05), except Avoidance of Death showed non-significant correlations with trait anxiety (*r* = 0.065, *p* = 0.230) and subjective happiness (*r* = 0.029, *p* = 0.587).

**Table 3:** Means, standardized deviation (SD) of measures, and correlations between overall of SDA, four dimensions of SDA and other measures.

	Depression^1^	IES-R intrusion	IES-R avoidance	IES-R hyperarousal	Trait Anxiety	Subjective Happiness
*Mean*	0.60	1.58	1.56	1.20	2.22	4.97
*SD*	0.41	0.76	0.79	0.76	0.38	1.04
*r* with						
Overall SDA	0.401^∗∗^	0.470^∗∗^	0.413^∗∗^	0.475^∗∗^	0.386^∗∗^	–0.244^∗∗^
*Dysphoria*	0.464^∗∗^	0.462^∗∗^	0.393^∗∗^	0.482^∗∗^	0.421^∗∗^	–0.293^∗∗^
*Death Intrusion*	0.357^∗∗^	0.357^∗∗^	0.256^∗∗^	0.330^∗∗^	0.333^∗∗^	–0.268^∗∗^
*Fear of Death*	0.259^∗∗^	0.367^∗∗^	0.356^∗∗^	0.379^∗∗^	0.253^∗∗^	–0.123^∗^ (*p* = 0.023)
*Avoidance of Death*	0.145^∗^ (*p* = 0.007)	0.145^∗^ (*p* = 0.007)	0.179^∗^ (*p* = 0.001)	0.154^∗^ (*p* = 0.004)	0.065 (*p* = 0.230)	0.029 (*p* = 0.587)

*^1^*n* = 341; ^∗∗^*p* < 0.001; *r* the coefficient of correlation between SDA and other criterion measures. SDA, Scale of Death Anxiety; Depression, measured by using Beck Depression Inventory II; IES-R, Impact of Event Scale-Revised.*

#### Effects of Gender, Age, and Religion on SDA

**Table 4** shows the effects of gender, religion, and age on the overall SDA and four dimensions of SDA. Independent *t*-tests showed no difference in the overall SDA and four dimensions of SDA between males and females, *t*(340) < 1.51, *p* > 0.13; the same was true of religious and non-religious, *t*(336) < 0.52, *p* > 0.60. However, older individuals reported a lower score on the overall SDA than younger individuals, *r* = -0.11, *p* = 0.05, 95% CI = [-0.208, -0.006] based on 1000 bootstrap samples. As **Table 4c** shows, older individuals perceived less fear of death than younger ones (*r* = -0.12, *p* = 0.02). Furthermore, multi-group confirmatory factor analyses showed that the invariance of the factor model’s parameters between gender (male vs. female), religious, and age are not significant. And these are reported in detail in the Supplementary Data Sheet 1 in the Supplementary Material.

**Table 4:** Effects of gender **(a)**, religion **(b)**, and age **(c)** on the overall and four dimensions of SDA.

		Overall SDA	Dysphoria	Death Intrusion	Fear of Death	Avoidance of Death
**(a)**

Male	*Mean*	2.14	2.22	1.83	2.22	2.41
	*SD*	0.69	0.92	0.81	1.04	0.93
Female	*Mean*	2.12	2.22	1.70	2.22	2.52
	*SD*	0.63	0.90	0.66	0.93	0.84
*t*(340)		0.25	–0.04	1.51	0.01	–1.08
*p*		0.80	0.97	0.13	0.99	0.28

**(b)**						

Religious	*Mean*	2.13	2.15	1.79	2.30	2.46
	*SD*	0.64	0.96	0.69	1.15	0.90
Non-religious	*Mean*	2.14	2.24	1.74	2.21	2.48
	*SD*	0.69	0.90	0.73	0.94	0.87
*t*(336)		0.06	–0.51	0.37	0.52	–0.12
*p*		0.95	0.61	0.71	0.60	0.90

**(c)**						

*r* with age		–0.11	–0.06	–0.07	–0.12	–0.07
*p*		0.05	0.29	0.19	0.02	0.22

Overall, the 17-item SDA with four factors demonstrated a good model fit in a second Chinese sample. Results showed good reliability, construct validity, and criterion validity of the SDA. The overall SDA as well as the four dimensions were positively associated with depression, trait anxiety, and the impact of stressful events, and negatively associated with subjective happiness, except for the behavioral component of the SDA: Avoidance of Death, with an alpha of 0.57, showed non-significant associations with the trait anxiety, and the subjective happiness. This non-significant relationship may be partly due to the small number of items (*n* = 3), but mostly because of the nature of the youth sample, whose psychological states are unstable and changeable. Therefore, future studies are needed to continue to test the four-factor model of the SDA in samples with different ages, especially for the behavioral component of SDA. The current findings also suggested a negative correlation between age and the SDA, especially for perceived fear of death, which was consistent with previous research (e.g., Rasmussen and Brems, 1996; Tang et al., 2002). The current study provided evidence of the relationship between age and death anxiety in a younger sample (ranging from 13 to 23 years old), indicating that the SDA is an age-sensitive measure.

Furthermore, there was a non-significant effect of gender and religion on the SDA. Although these findings were consistent with some previous research (Neimeyer, 2009), there is one possible explanation for the results in the current study. The age of the current sample ranged from 13 to 23 years old, which is younger than the samples that showed a gender difference. For example, Russac et al. (2007) found that women experienced higher death anxiety in their 1950s. With respect to the non-significant effect of religion on the SDA in the current sample, this is reasonable due to the younger sample we used. Current non-significant findings suggest that religion could not predict youth perception of death anxiety. However, considering the large gap between the two subgroups (33 had a religion vs. 305 no religion), future studies are needed to further explore this interesting topic. Considering the complicated influence of religion, we propose an open and complicated relationship of religion and death anxiety, and situational factors (such as uncontrollable disasters) may influence this link.

## General Discussion

The 17-item SDA is a reliable and valid measure of individual differences in and perceptions of death anxiety. Following a systematic process, a four-factor structure of the SDA was identified that revealed four aspects of death anxiety: Dysphoria, Death Intrusion, Fear of Death, and Avoidance of Death. The results of this study indicate that the SDA has a clear factor structure and good psychometric properties in Chinese samples. The SDA supports death anxiety as a multidimensional construct, and the foundational role of fear of death in the generation of death anxiety. This scale is valuable and beneficial to research on death anxiety.

The SDA reflects both the nature of anxiety and death. The four dimensions of the SDA represented the death anxiety in terms of somatic, cognitive, emotional, and behavioral aspects. Death anxiety is a kind of anxiety particular to one’s own death. Not like other dreadful events or stimulation, death is far away from most individual daily life, is certain to exist, and cannot be adjusted. Differing from other measurements of death anxiety, the SDA measures (1) the dysphoria and somatic components of death anxiety on individuals, which reflects the nature of anxiety; (2) the general emotional and behavioral components of death anxiety when thinking of one’s own death, and not other specific related matters (such as tombs); (3) the intrusive phenomenon of one’s own death from a symptomatic perspective.

The SDA contributes significantly not only by providing a valuable tool that measures components of death anxiety, but also by examining death anxiety from a symptomatic perspective. As described in the “Materials and Methods” section, the items of the SDA were generated based on both previous literature and the theoretical definition of death anxiety. We also used measures of anxiety as references and chose items from a symptomatic perspective. Furthermore, the SDA measures individual perceptions and feelings of death anxiety in the past month, because if a symptom lasts more than 1 month, it may reach the clinical criterion for a psychological disorder. In addition, the SDA aims to measure death anxiety in the general population, not those with a clinical diagnosis. Therefore, the SDA focuses on individuals’ perceived death anxiety in the past month.

Referring to the four specific dimensions of the SDA, to our knowledge, this is the first time that Dysphoria has been measured in a scale for death anxiety. This component reflects the nature of anxiety and directly represents physiological symptoms experienced by individuals when they think of death. The results of the PCA in Study 1 showed the largest eigenvalue for Dysphoria, which indicates this factor could explain the most score variation of the full SDA among the four factors.

Apart from the novel Dysphoria component in the scale, the SDA also measured Fear of Death and Avoidance of Death. Both are accepted by other researchers and are consistent with previous instruments (e.g., Nelson and Nelson, 1975; Nelson, 1978). In the SDA, four items comprise the Fear of Death factor, reflecting both emotional (e.g., “…*felt scared*”) and somatic (e.g., “…*my heart beat fast*”) reactions caused by death anxiety, and three items comprised the Avoidance of Death factor, reflecting cognitive (e.g., “…*avoided thoughts or ideas related to death*”) and behavioral (e.g., “…*avoided events or situations related to death*”) symptoms derived from death anxiety. These factors do not include items about fear of specific death-related events, which differs from previous instruments (e.g., Templer, 1970; Abdel-Khalek and Lester, 2004). We believe that all of these specific fears (e.g., of tombs, dead bodies, or illness) are caused by the deep fear of one’s own death. Therefore, the SDA uses more general and direct words about death to measure Fear of Death, or general death anxiety, to avoid problems associated with variation in death-related events across cultures.

Last, Death Intrusion of the SDA measures intrusive nightmares, imagery, and thoughts related to one’s own death. In terms of theoretical enlightenment, the core belief of death anxiety is the apprehension of one’s own death (e.g., Yalom, 1980; Freud, 1914). Death Intrusion of the SDA directly reflects an individual’s recurrent thoughts of death, which are both a cause and consequence of death anxiety. However, Death Intrusion has not been seriously considered in previous measures (e.g., Lonetto and Templer, 1986).

Apart from enriching our understanding of death anxiety and benefitting researchers conducting further investigations in this field, the SDA is useful in clinical practice. Based on a symptomatic perspective, the SDA builds a potential link between clinical and counseling diagnoses and psychological assessment. Taking the relationship of death anxiety and mental disorders (Arndt et al., 2005; Strachan et al., 2007) into consideration, the SDA may enable comprehension of related psychological disorders and identification of effective therapies. Apart from these obvious contributions, it is important to note that this is the first study to explore the dysphoria and somatic symptoms of death anxiety, that the SDA was developed using limited samples, and that it is not clear whether this measure of death anxiety is invariant across cultures, time, and age. Therefore, it is necessary and important to replicate the four-factor structure of the SDA in other samples with different cultures. Moreover, future studies are needed to continue exploring the reliability and validity of this instrument in larger samples with different cultures, ages, and religions. Second, the current study did not test the associations between death anxiety and psychological disorders, such as panic disorder, that are characterized by strong anxiety symptoms. Considering evidence that death anxiety has been associated with some psychological disorders (Arndt et al., 2005; Strachan et al., 2007), we assume a positive relationship between the SDA and such psychological disorders. Future studies are needed to test this assumption.

Taken together, a new measure of death anxiety (i.e., SDA) was developed and validated in two Chinese youth samples. To our knowledge, this is the first instrument of death anxiety that includes dysphoria and somatic symptoms. The SDA was reliable and valid for the current samples with a four-factor structure: Dysphoria, Death Intrusion, Fear of Death, and Avoidance of Death. Finally, the 17-item SDA is simple and a reasonable length to measure death anxiety. We believe that the SDA is a valuable reliable and valid measure in the study of death anxiety.

## Author Contributions

WC and Y-lT constructed the original idea and concepts; WC performed studies and draft the manuscript. WC, SW, and HL analyzed data. Y-lT, SW, and HL critically revised the paper. All authors approved the final version of the manuscript for submission.

## Conflict of Interest Statement

The authors declare that the research was conducted in the absence of any commercial or financial relationships that could be construed as a potential conflict of interest.
