# Supplementary material for: Scale of Death Anxiety (SDA): Development and Validation
Source: Front Psychol. 2017 May 31;8:858. doi: 10.3389/fpsyg.2017.00858 (PMC5449485; doi:10.3389/fpsyg.2017.00858)
Supplement: Supplementary file 1 [file Data_Sheet_1.pdf]

Supplemental Material

MGCFA for male

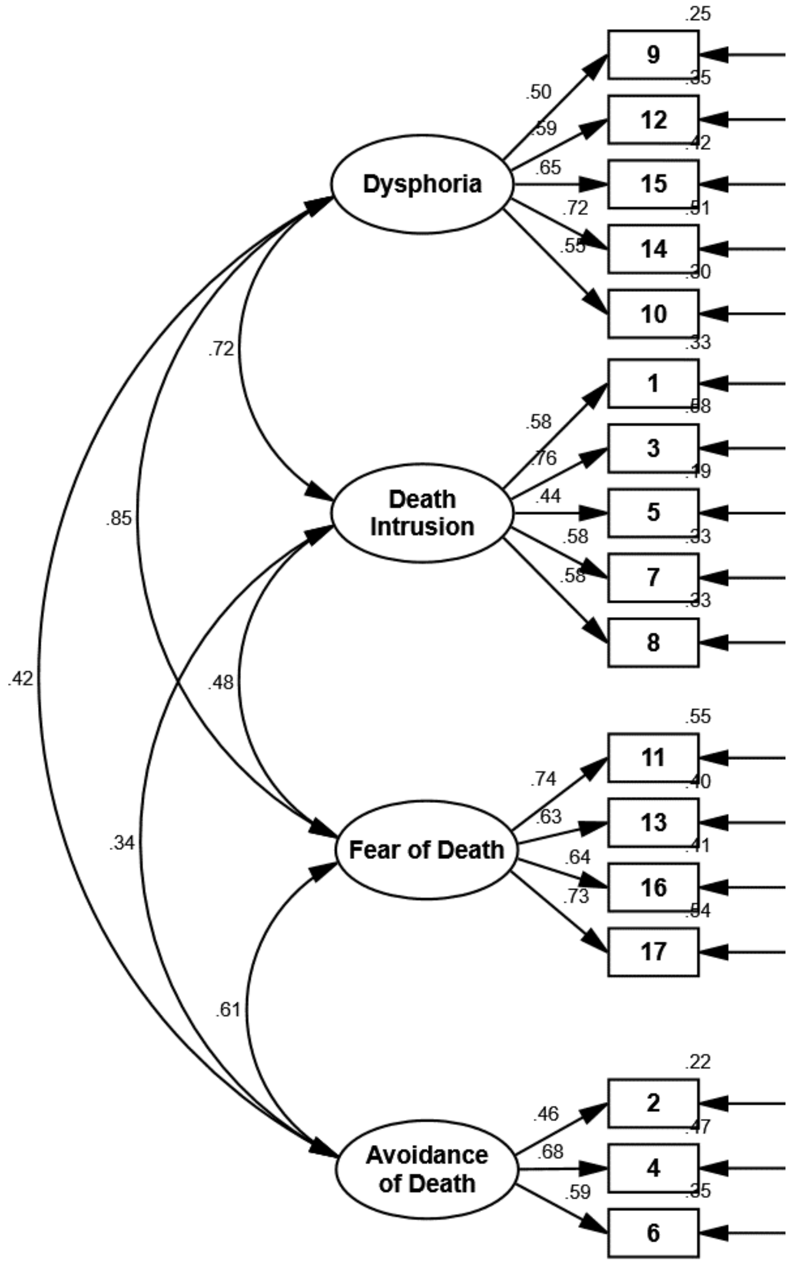

For male, n = 116, chi^2 = 203.36, chi^2/df = 1.83, p < .001, CFI = .85, RMSEA = .08, SRMR = .089.

MGCFA for female

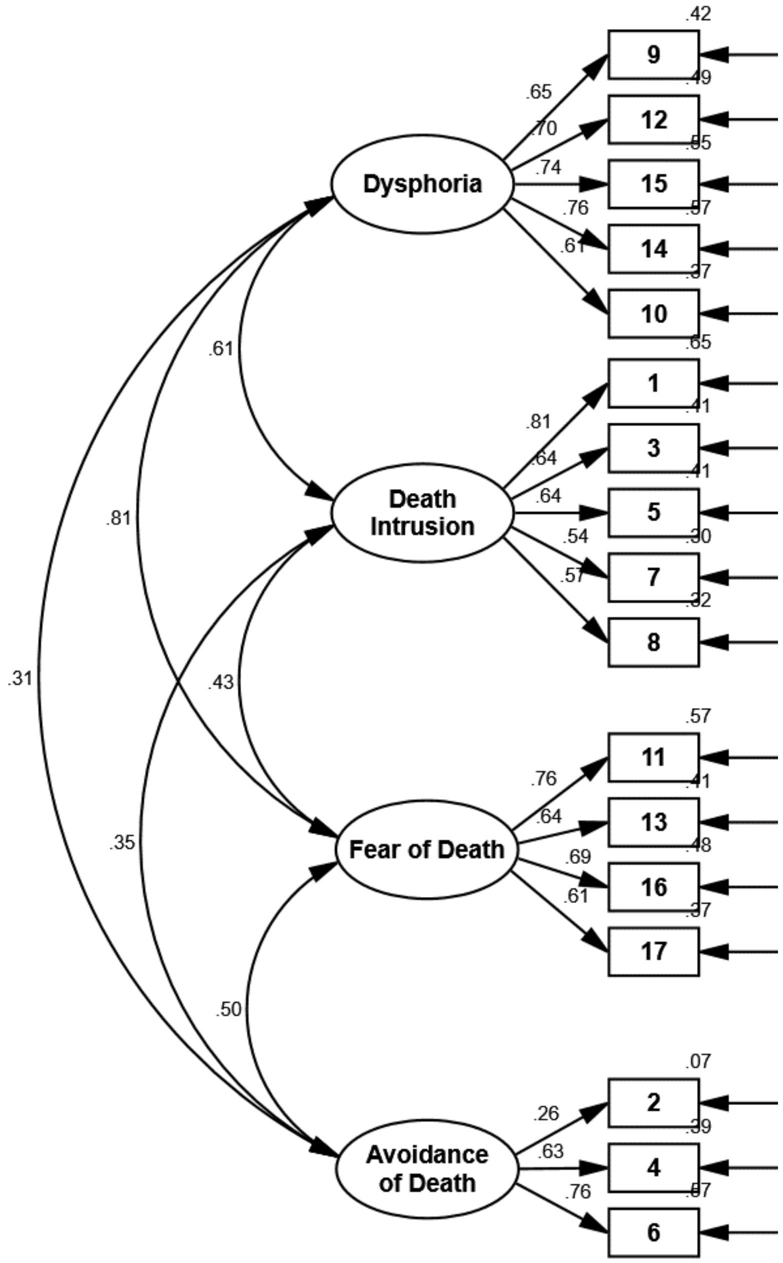

For female, n = 226, chi^2 = 229.58, chi^2/df = 2.07, p < .001, CFI = .91, RMSEA = .069, SRMR = .064.

MGCFA for non-religious

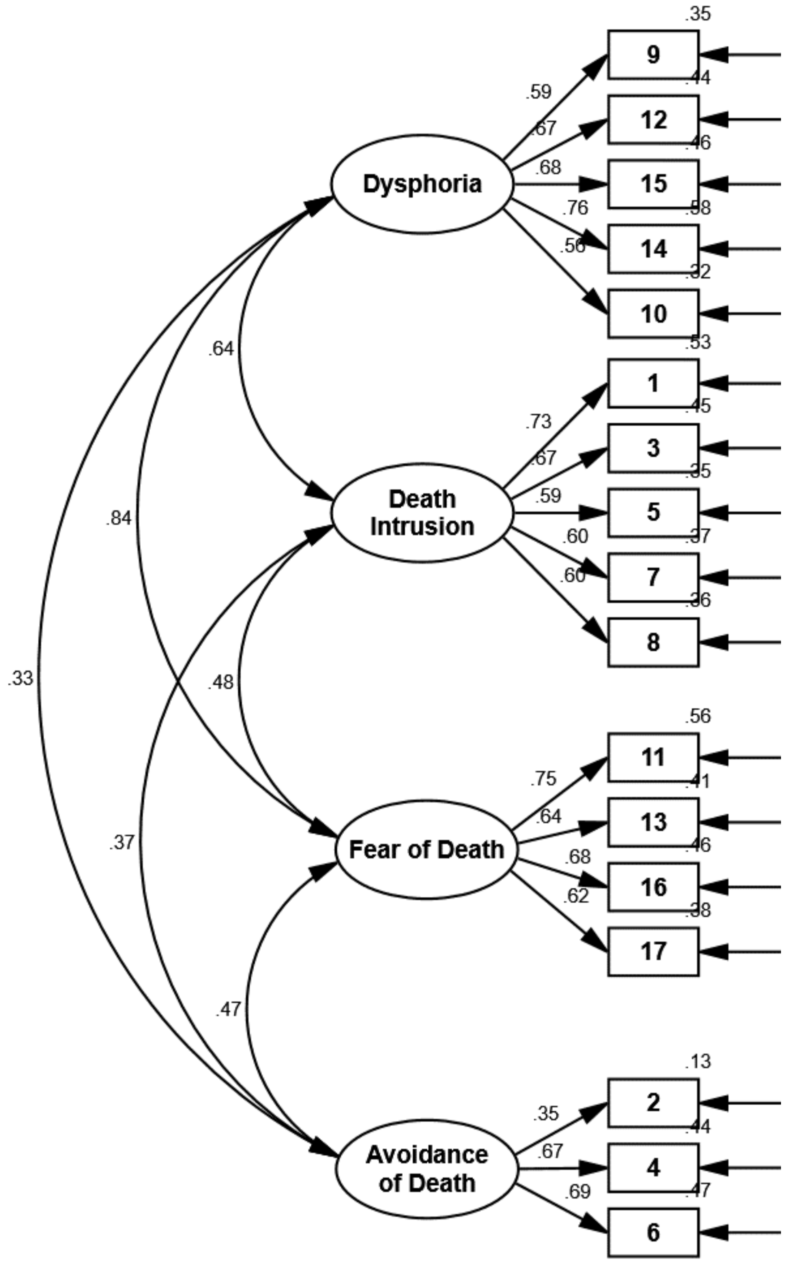

For non-religious, n = 305, chi^2 = 317.78, chi^2/df = 2.86, $p < .001$, CFI = .88, RMSEA = .078, SRMR = .063.

MGCFA for age (1 = age <17, n = 75; 2 = age >=17, n = 267) only

condition 2, age > =17 worked.

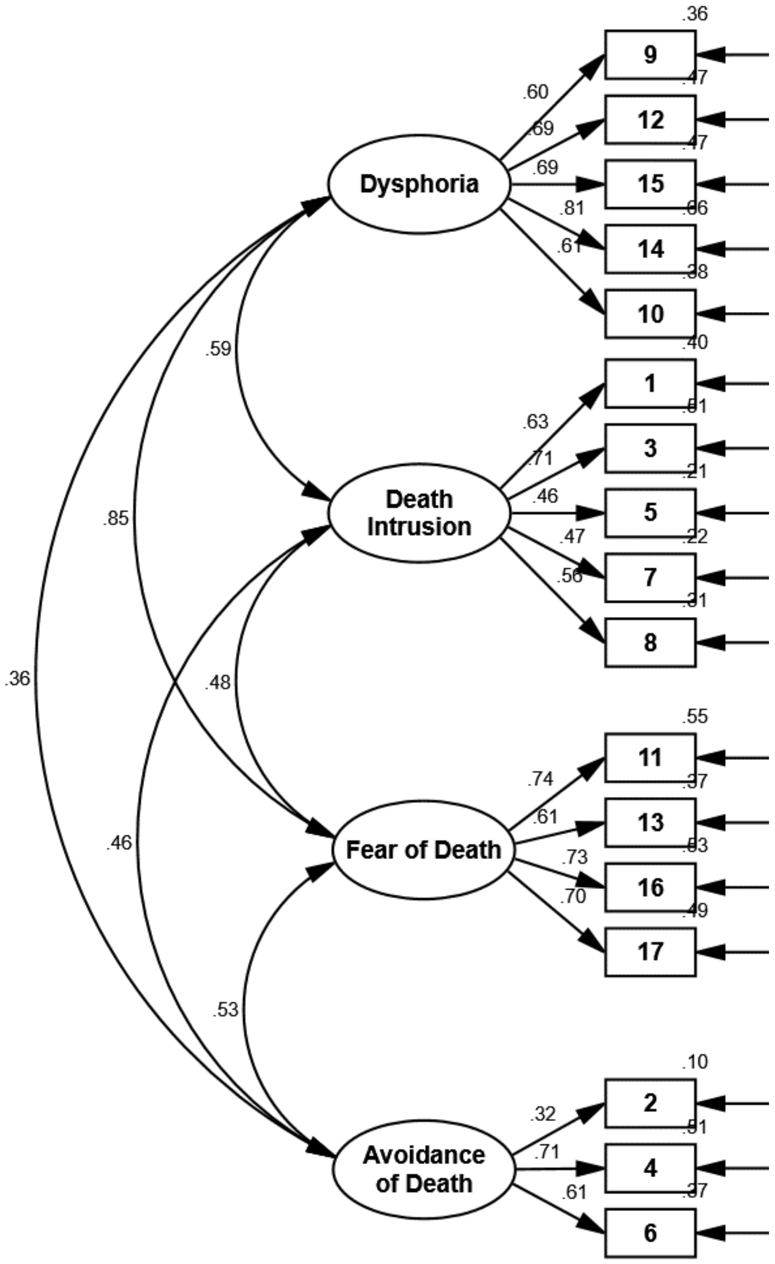

For adults (age > 16), n = 267, $chi^2 = 243.95$, $chi^2/df = 2.20$, $p < .001$, CFI = .91, RMSEA = .067, SRMR = .057.
